# Effects of salvianolic acid B on liver fibrosis: A protocol for systematic review and meta analysis

**DOI:** 10.1097/MD.0000000000021036

**Published:** 2020-07-10

**Authors:** Xiaocong Ma, Meiwen Tang, Liying Lu, Jinghui Zheng, Jingjing Huang, Junhong Li, Weisheng Luo

**Affiliations:** a Graduate School, Guangxi University of Chinese Medicine; b Department of Geriatrics, Ruikang Hospital Affiliated to Guangxi University of Chinese Medicine; c The First Affiliated Hospital of Guangxi University of Chinese Medicine; d Department of Gastroenterology, Ruikang Hospital Affiliated to Guangxi University of Chinese Medicine, Nanning, Guangxi, China.

**Keywords:** liver fibrosis, meta-analysis, salvianolic acid B, study protocol, systematic review

## Abstract

**Background:**

Liver fibrosis is a pathological change existing in most chronic liver diseases, which leads to abnormal changes in liver tissue structure and affects the normal physiological function of liver. Without effectively control, liver fibrosis can develop into cirrhosis and increase the risk of liver cancer. Salvianolic acid B (Sal B) is the main active component in the water-soluble extract from Salvia miltiorrhiza, which is a traditional Chinese medicine usually used for treating cardiovascular and liver diseases. It is reported that Sal B shown a good action against liver fibrosis via numerous signaling pathways, which indicate that Sal B is a potential candidate drug for the treatment of liver fibrosis.

**Methods:**

We searched the related researches from the following electronic databases: PubMed, EMBASE, Web of science, China National Knowledge Infrastructure (CNKI), China Biology Medicine (CBM), Wan fang Database for Chinese Technical Periodicals and VIP Database. All the databases were searched from inception to December 2019. No restriction of language, publication date, or publication status. PICO of this systematic review are shown as flowing: P, preclinical studies which evaluated the effects of Sal B on the animal models of liver fibrosis with controlled studies; I, received Sal B as only treat in any dose; C, received normal saline, distilled water, or no treatment; O, the primary outcome include measure will be the decrease in liver fibrosis score, and the secondary outcomes include the index of liver fibrosis. All the included data will be analyzed with the software of Review Manager 5.2 and STATA 14.2.

**Discussion:**

The purpose of this study is to conduct a systematic review and meta-analysis to assess the effects on anti-liver fibrosis of Sal B, and this will be contribute to drug development and pathological mechanisms of clinical research.

**Trial registration:**

INPLASY202050101, registered on 28/5/2020.

## Abstract 2

Supplemental Digital Content is available in the text

## 1 Introduction

Liver fibrosis is a pathological change existing in most chronic liver diseases, mainly manifested as the excessive hyperplasia and deposition of the extracellular matrix (ECM) in liver tissues, which leads to abnormal changes in liver tissue structure and affects the normal physiological function of liver.^[1]^ Without effectively control, liver fibrosis can develop into cirrhosis and increase the risk of liver cancer.^[2]^ In fact, liver fibrosis is a common result of abnormal wound healing of chronic liver injury caused by a variety of etiologies and hoping to be reversed and cured.^[3]^ The annual incidence of chronic hepatitis B progression to cirrhosis is 2% to 10%.^[4]^ Therefore, active treatment of liver fibrosis to reverse or delay its development is of great significance to improve the quality of life of patients and improve the prognosis of the disease.

At present, the treatment of liver fibrosis mainly includes eliminating stimulation or harmful factors, inhibiting liver inflammation, interfering with the activation of stellate cells, and promoting the deterioration of ECM.^[5]^ If treated timely, liver fibrosis and early stage cirrhosis can be reversed.^[6,7]^ However, the effect of western medicine treatment of liver fibrosis is not ideal because it lead to a series of adverse reactions, coupled with the complex pathophysiological of liver fibrosis, there is still lacks effective western medicine in clinical.^[8,9]^ It is noteworthy that the active ingredients in Traditional Chinese Medicine are reported to have anti-hepatic fibrosis effects.^[10]^

Salvianolic acid B (Sal B) is the main active component in the water-soluble extract from Salvia miltiorrhiza, which is a traditional Chinese medicine usually used for treating cardiovascular and liver diseases.^[11,12]^ Sal B have various pharmacological effects, such as antagonizing atherosclerosis, alleviating myocardial ischemia/reperfusion injury, anti-inflammation and alleviation liver injury.^[13]^ It is reported that Sal B shown a good action against liver fibrosis in animal model experiment via numerous signaling pathways,^[14,15]^ which indicate that Sal B is a potential candidate drug for the treatment of liver fibrosis. However, the efficacy of Sal B for liver fibrosis have not been systematically evaluated.

To be the best knowledge, the purpose of this study is to conduct a systematic review and meta-analysis to assess the effects on anti-liver fibrosis of Sal B, and this will be contribute to drug development and pathological mechanisms of clinical research.

## 2 Methods

### 2.1 Design

The review authors conducted this systematic review and meta-analysis protocol according to the Preferred Reporting Items for Systematic Review and Meta-analysis Protocol Guidelines (PRISMA-P)^[16]^ and the recommendations for reporting of systematic reviews and meta-analyses of animal experiments.^[17,18]^ The protocol was registered on International Platform of Registered Systematic Review and Meta-analysis Protocols (INPLASY202050101).

### 2.2 Eligibility criteria

#### 2.2.1 Types of studies

The systematic review will include preclinical studies which evaluated the effects of Sal B on the animal models of liver fibrosis with controlled studies. No restriction of language, date, or publication status.

#### 2.2.2 Types of animal models

We will include the animal models of liver fibrosis and which histopathology proved that there were liver fibrosis in animal model.

#### 2.2.3 Types of intervention

The intervention group will include animals that treatment with Sal B (compounds extracted from Salvia miltiorrhiza) and received Sal B as only treat in any dose, the Sal B have been administered during or after the induced of liver fibrosis animal models.

#### 2.2.4 Types of comparators

The comparison group will include animals that interventions with normal saline, distilled water, or no treatment.

#### 2.2.5 Types of outcome measures

Primary outcome include measure will be the decrease in Liver Fibrosis Score, evaluated through histopathologic examination, and measured at the last time following administration of the intervention. Secondary outcomes include the index of liver fibrosis, such as HA, LN, PCIII, Hyp; index of liver function, such as ALT, AST, ALB, TBIL; indicators of oxidative stress, such as SOD, MDA, and GSH.

### 2.3 Exclusion criteria

In this systematic review, vitro experiment, abstract of congress, letter to the editor, and human studies will be excluded; Sal B conjunction with other compounds or Sal B based prescriptions will be excluded because effect size may not be due to salvianolic acid B. Non-liver Fibrosis model, no control group, duplicate publications, and no available data also will be excluded.

### 2.4 Information source

#### 2.4.1 Electronic searches

The systematic review will searched from the following electronic databases: PubMed, EMBASE, Web of science, China National Knowledge Infrastructure (CNKI), China Biology Medicine (CBM), Wan fang Database for Chinese Technical Periodicals, and VIP Database. All the databases were searched from inception to December 2019. No restriction of language, publication date, or publication status.

#### 2.4.2 Searching other resources

We will screen the sites of animal research organizations, Google Scholar, Baidu Scholar. In manual search, 2 reviewers will examine the reference list or citations found in the secondary studies to validate and identify possible eligible studies.

The search strategy will use a combination of MESH terms and free terms. Details of the PubMed search strategy appear in Supplementary Materials 1.

### 2.5 Selection of studies

**Figure 1:**
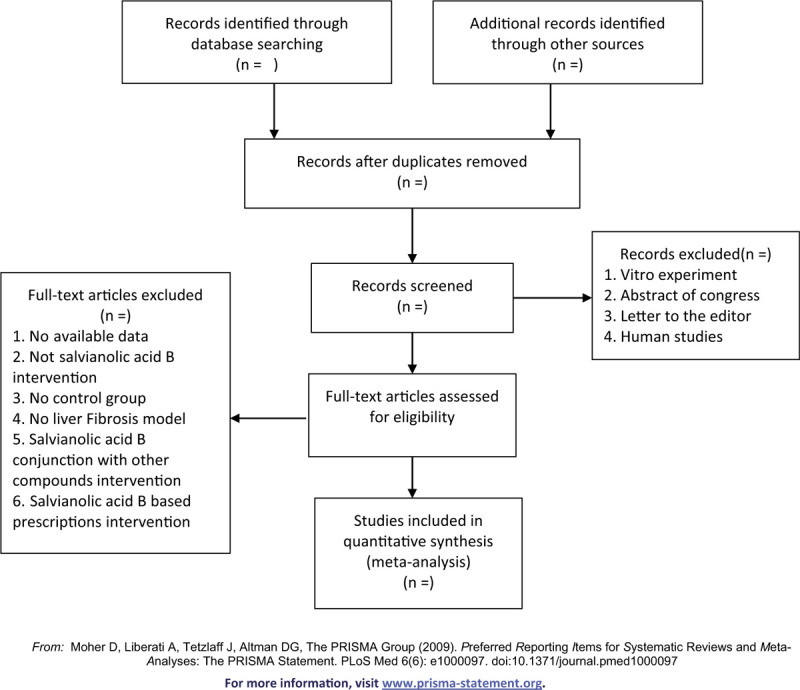
Screening process.

All the studies will imported to the EndNote software of version X9. Two independent authors will screen the titles and abstracts. The duplicates studies will be removed during the screening process. Disputes between authors will be discussed and resolved by group discussion or third review. After initial screen and de-duplication, the same 2 independent authors will estimate the full text of studies according to the included and excluded criteria. For the duplicate publication studies, we will select the studies which with more complete data. The screening process was shown as Figure 1.

### 2.6 Data extraction

The same 2 independent authors extracted the data following details from included studies:

1. the first authors name and publication year, model of liver fibrosis, and the anesthesia methods for model preparation;
2. the specific information of animals for each study, including animal species, number, sex, and weight;
3. the treatment groups information, including therapeutic drug dosage, method of administration, duration of treatment, and the same information of control group;
4. the outcomes’ data of mean value and standard deviation were extracted from every study, including liver fibrosis score, index of liver fibrosis (HA, LN, PCIII, Hyp) and index of liver function (ALT, AST, ALB, TBIL), index of oxidative stress (SOD, MDA, GSH), and timing for outcome assessments.

The data of highest dose was included when the treatment group included various doses of the target drug. The result of the peak time point was included when the data were expressed at different times.

### 2.7 Risk of bias and quality assessment

The methodological quality of the included studies will be assessed according to the SY stematic Review Centre for Laboratory animal Experimentation SYRCLEs risk of bias tool, and the Collaborative Approach to Meta-Analysis and Review of Animal Data from Experimental Studies (CAMARADES) checklist that evaluate the included studies, 1 point was given for each of the following criteria:

1. peer reviewed publication;
2. control of temperature;
3. random allocation to treatment or control;
4. blinded induction of model;
5. blinded assessment of outcome;
6. use of anesthetic without significant intrinsic neuroprotective activity;
7. animal model (aged, diabetic, or hypertensive);
8. sample size calculation;
9. compliance with animal welfare regulations;
10. statement of potential conflict of interests.

### 2.8 Measure of treatment effect and data analysis

All the included data will be analyzed with Review Manager 5.2 version software (The Cochrane Collaboration, Software Update, Oxford, United Kingdom). The continuous or dichotomous type of date will be included in this review, and the estimate of the continuous date combined effect sizes will calculate by the standardized mean difference (SMD), Dichotomous data will be calculated by the odds ratio (OR), 95% confidence intervals (CIs) of all results were calculated.

### 2.9 Heterogeneity assessment

The heterogeneity will be checked by *I*^2^ statistics, when *I*^2^ statistics is higher than 50%, indicates a high heterogeneity, random effects model will be used for meta-analysis; otherwise fixed effects model will be carried out. If there is high heterogeneity, we will find the potential sources of heterogeneity by using the methods of sensitivity analysis and subgroup analysis. If the data is not suitable for pooled or subgroup analysis, we will conducted a narrative description analysis of the findings. If the data are available, subgroup analysis will be performed with the following groups: animal species, the method of animal model induced, and the duration of treatment.

### 2.10 Publication bias assessment

The publication bias will be evaluated (no less than 10 studies contribute to the summary analysis) by using the funnel plots combine with statistical test (Egger and Begg test), and the Egger and Begg test will be performed by STATA software with version 14.2 (Stata Corp, College Station, USA).

## 3 Discussion

The purpose of this systematic review and meta-analysis is to provide preclinical research evidence and existing relevant evidence regarding Sal B to improve the fibrosis degree in animal models of liver fibrosis. At the same time, this study aim to show the advantages and limitations of the current literature, and put forward the future research prospects in this field.

Although many experimental studies on the effect of Sal B on liver fibrosis have been published, but there is no consensus in the literature. Therefore, it is necessary to conduct a systematic review of the existing experimental studies.

## Author contributions

Xiaocong Ma designed this research and drafted the manuscript, Meiwen Tang tested the feasibility of the study, Liying Lu and Jinghui Zheng contributed to the development of the selection of studies and data extraction, Jingjing Huang contributed to risk of bias and quality assessment, Junhong Li conducted the data analysis and Weisheng Luo read, provided feedback and approved the final manuscript. All authors approved the final version of the manuscript.

## Supplementary Material

Supplemental Digital Content

## References

[1] Yoon YJ Friedman SL Lee YA Antifibrotic therapies: where are we now? Semin Liver Dis 2016;36:87–98. 26870935 10.1055/s-0036-1571295

[2] Kocabayoglu P Friedman SL Cellular basis of hepatic fibrosis and its role in inflammation and cancer. Front Biosci (Schol Ed) 2013;5:217–30. 23277047 10.2741/s368

[3] Lee YA Wallace MC Friedman SL Pathobiology of liver fibrosis: a translational success story. Gut 2015;64:830–41. 25681399 10.1136/gutjnl-2014-306842 PMC4477794

[4] Fattovich G Bortolotti F Donato F Natural history of chronic hepatitis B: special emphasis on disease progression and prognostic factors. J Hepatol 2008;48:335–52. 18096267 10.1016/j.jhep.2007.11.011

[5] Altamirano-Barrera A Barranco-Fragoso B Méndez-Sánchez N Management strategies for liver fibrosis. Ann Hepatol 2017;16:48–56. 28051792 10.5604/16652681.1226814

[6] Chen X Li WX Chen Y Suppression of SUN2 by DNA methylation is associated with HSCs activation and hepatic fibrosis. Cell Death Dis 2018;9:1021. 30282980 10.1038/s41419-018-1032-9 PMC6170444

[7] Xu T Ni MM Xing L NLRC5 regulates TGF-(1-induced proliferation and activation of hepatic stellate cells during hepatic fibrosis. Int J Biochem Cell Biol 2016;70:92–104. 26592197 10.1016/j.biocel.2015.11.010

[8] Wang JN Li L Li LY Emerging role and therapeutic implication of Wnt signaling pathways in liver fibrosis. Gene 2018;674:57–69. 29944952 10.1016/j.gene.2018.06.053

[9] Wu Y Liu X Zhou Q Silent information regulator 1 (SIRT1) ameliorates liver fibrosis via promoting activated stellate cell apoptosis and reversion. Toxicol Appl Pharmacol 2015;289:163–76. 26435214 10.1016/j.taap.2015.09.028

[10] Shan L Liu Z Ci L Research progress on the anti-hepatic fibrosis action and mechanism of natural products. Int Immunopharmacol 2019;75:105765. 31336335 10.1016/j.intimp.2019.105765

[11] Chen W Chen G Danshen (Salvia miltiorrhiza Bunge): a prospective healing sage for cardiovascular diseases. Curr Pharm Des 2017;23:5125–35. 28828985 10.2174/1381612823666170822101112

[12] Li S Wang N Hong M Hepatoprotective effects of a functional formula of three chinese medicinal herbs: experimental evidence and network pharmacology-based identification of mechanism of action and potential bioactive components. Molecules 2018;23:352. 10.3390/molecules23020352 PMC6017312 29414910

[13] Cao W Guo XW Zheng HZ Current progress of research on pharmacologic actions of salvianolic acid B. Chin J Integr Med 2012;18:316–20. 22457144 10.1007/s11655-012-1052-8

[14] Tsai MK Lin YL Huang YT Effects of salvianolic acids on oxidative stress and hepatic fibrosis in rats. Toxicol Appl Pharmacol 2010;242:155–64. 19822164 10.1016/j.taap.2009.10.002

[15] Wang R Yu XY Guo ZY Inhibitory effects of salvianolic acid B on CCl(4)-induced hepatic fibrosis through regulating NF-(B/I(B( signaling. J Ethnopharmacol 2012;144:592–8. 23041223 10.1016/j.jep.2012.09.048

[16] Shamseer L Moher D Clarke M PRISMA-P Group,. Preferred reporting items for systematic review and meta-analysis protocols (PRISMA-P) 2015: elaboration and explanation. BMJ 2015;349 (jan02 1):g7647. 10.1136/bmj.g7647 25555855

[17] Sena ES Currie GL McCann SK Systematic reviews and meta-analysis of preclinical studies: why perform them and how to appraise them critically. J Cereb Blood Flow Metab 2014;34:737–42. 24549183 10.1038/jcbfm.2014.28 PMC4013765

[18] Peters JL Sutton AJ Jones DR A systematic review of systematic reviews and meta-analyses of animal experiments with guidelines for reporting. J Environ Sci Health B 2006;41:1245–58. 16923604 10.1080/03601230600857130

