# Supplementary material for: Effects of salvianolic acid B on liver fibrosis: A protocol for systematic review and meta analysis
Source: Medicine (Baltimore). 2020 Jul 10;99(28):e21036. doi: 10.1097/MD.0000000000021036 (PMC7360269; doi:10.1097/MD.0000000000021036)
Supplement: Supplemental Digital Content [file medi-99-e21036-s001.docx]

[Liver Cirrhosis(MESH) OR Hepatic Fibrosis (Title/Abstract) OR Hepatic Cirrhosis (Title/Abstract) OR Liver Fibrosis (Title/Abstract)] AND [salvianolic acid B (MESH)]
